# Sustaining grant-funded programs: Conditions for sustaining initiatives that enhance diversity in STEMM

**DOI:** 10.1371/journal.pone.0343368

**Published:** 2026-02-20

**Authors:** Naomi A. Stephen, Krystle P. Cobian, Ana L. Romero, Hector V. Ramos, Sylvia Hurtado

**Affiliations:** 1 School of Education and Information Studies, University of California, Los Angeles, California, United States of America; 2 David Geffen School of Medicine, University of California, Los Angeles, California, United States of America; 3 University of Texas, El Paso, Texas, United States of America; Institute of Medical Biochemistry Leopoldo de Meis (IBqM) - Federal University of Rio de Janeiro (UFRJ), BRAZIL

## Abstract

As funding agencies seek to broaden their impact in science, technology, engineering, mathematics, and medicine (STEMM) and workforce diversity, many grants have required plans for sustaining institutional change beyond the grant-period. However, little is known about the types of STEMM interventions that are likely to be sustained. Employing a multiple case study design, we examine the NIH BUilding Infrastructure Leading to Diversity (BUILD) initiative, at 10 awarded higher education sites nationwide, designed to promote sustainable change in biomedical workforce diversity. Among all the activities developed by program sites, undergraduate research training, faculty mentoring training, and curricular changes were most likely to be sustained in the final two years of the 10-year award period. Drawing on implementation science, we also examined why interventions were sustainable and identified four contextual elements that largely facilitated program sustainability: institutional financial status; organizational infrastructure and partnerships; central administration and STEMM faculty commitment; and alignment with institutional priorities. Ultimately, this study provides key lessons for future grant-funded teams and senior administrators engaged in efforts to promote equity and inclusion in STEMM.

## Introduction

As demographic shifts continue to occur across the educational landscape, national goals remain salient for diversifying the biomedical workforce and maintaining an international scientific edge in science, technology, engineering, mathematics, and medicine (STEMM). Several National Academies of Sciences, Engineering, and Medicine (NASEM) reports study the rationale for cultivating diverse talent and recommend reimagining institutions, developing organizational and educational support, and extending funding to under-resourced institutions and individuals along the STEMM pathway (e.g., [1–3]). Since historically minoritized racial and ethnic groups will compose the majority of the U.S. population before 2050 [4], transforming STEMM education pathways has become paramount. This is the significant backdrop that initiated and fueled one of the most historic and comprehensive investments of the National Institutes of Health (NIH) in STEMM workforce diversity, featuring 10-year institutional grants focused on developing programs to increase and sustain the diversity of the NIH-funded workforce [5].

Despite growth in educational efforts to build workforce diversity, little research has been done to understand the efficacy and sustainability of educational initiatives aimed at transforming training in STEMM. Following a substantial investment, what elements of those grant-funded initiatives are maintained once external support has ended? Have they become embedded in the permanent infrastructure of higher education institutions to continue to meet both institutional aims and national goals? Without understanding how specific programs are maintained, we cannot understand how to implement future programs that will create long-term change. Institutionalizing and sustaining specific initiatives on campus has become even more important as the federal support was abruptly cut on programs focused on diversifying the future STEMM workforce in Spring 2025, forcing directors to seek other ways to support aspiring scientists [6].

### Background and overview of the BUILD Initiative

While the NIH funds institutional training programs to increase the numbers and skills of scientists who eventually seek and obtain NIH research grants, few programs have been rigorously evaluated at the institutional level. With this need in mind, in 2014, the NIH funded the *Enhance Diversity Study (EDS)* and the Diversity Program Consortium (DPC), which included the Coordination and Evaluation Center (CEC), BUilding Infrastructure Leading to Diversity (BUILD), and National Research Mentoring Network (NRMN). A primary aim of the EDS was to learn “what works, for whom, in what context, and why” [7]. To do so, the CEC was charged with evaluating the BUILD programs, individually and collectively [3 (p.111)].

BUILD is a historic research training and institutional capacity-building initiative that differs from prior NIH-funded training grants with its emphasis on evaluation and multi-pronged approach to enact change at the student, faculty, and institutional level [7,8]. The NIH selected 10 undergraduate institutions across the United States as primary sites for the BUILD awards. Institutional eligibility for funding included having less than $7.5 million in total NIH research funding and at least 25% enrollment of Pell Grant recipients. Each BUILD awardee proposed and implemented comprehensive site-specific approaches to engage students from diverse backgrounds in biomedical research, develop faculty mentoring and research skills, and promote institutional capacity to train biomedical researchers [7,8].

Another unique aspect of BUILD is the size and scope of the initiative. The awards provided over $350 million of funding, with over 3,000 students and over 4,000 faculty involved in the activities across the consortium of BUILD sites [7]. Each site implemented a variety of student- and faculty-centered initiatives involving evidence-based practices that have worked in other contexts but were novel innovations in these diverse campus contexts. After the first five years of funding, each of the BUILD awardees successfully applied for an additional five years of funding. During this second phase, grant funding was incrementally reduced each year (RFA-RM-18–006, 2018), and each awardee was required to develop plans to sustain program activities.

In order to accomplish the aim of the EDS, implementation science—the study of how evidence-based interventions are translated into practice [9]—is useful for examining the BUILD initiative and the ongoing longevity of practices. Our study identifies the implementation of various initiatives that moved from novel intervention to institution-wide practices and offers examples that can inform many STEMM interventions sponsored by federal and private foundations about the program characteristics and conditions for institutionalization and sustainability of initiatives. We focus on institutional context, a key concept from implementation science [10], to better understand how institutional conditions can facilitate program longevity. The theory that guides this work in implementation science posits that conditions are important for creating avenues for sustaining these efforts [11].

The purpose of this study was to examine and document efforts toward institutionalization and sustainability accomplished through the NIH-funded BUILD awards. Through a multiple case study design, we learned what BUILD sites accomplished at an institutional level and plan to maintain beyond grant funding. The following research questions guided this study:

1. *What kinds of initiatives aimed to enhance and maintain diversity and inclusion in biomedical sciences will be sustained and/or institutionalized after award funding ends?*
2. *What institutional conditions aided or inhibited program sustainability?*

## Literature review and framework

This section provides an overview of STEMM intervention programs (SIPs) that enhance diversity in STEMM as well as the literature on institutionalization. We then address implementation science literature derived from public health interventions to develop the foundation for understanding how contexts facilitate program institutionalization and sustainability.

### STEMM intervention programs

STEMM intervention programs (SIPs) that broaden participation of diverse populations were ubiquitous in higher education [7]. SIPs frequently have similar initiatives that include, for example, undergraduate research, financial support for students, faculty training, mentoring, and curricular change [12]. Initially, SIPs attempted to improve outcomes for students rather than institutional transformation. Early interventions included informal meetings to discuss classroom climate and how to improve outcomes for women [13]. After years of intensive focus on students, SIPs have taken on a much larger role–expanding the target populations and aiming to foster long-term change and transformation, such as focusing on how the institution facilitates student success. Palid et al. [14] argue that many SIPs have not met their objectives due to institutional failures, such as an unwelcoming environment for students and an overly complex financial and curricular landscape. Little research, though, explores whether additional funding or other conditions are needed to overcome institutional barriers [15].

Overall, the literature on SIPs has identified key program components that are now compulsory or highly encouraged for newer interventions [16,17]. SIPs that include mentoring and research participation have shown great success [14,18]. For example, several studies have found a strong positive association between high levels of faculty mentoring and student intent to pursue a science career [1,17,19,20]. Moreover, research participation has been identified as a positive predictor for a host of positive outcomes, including science identity, intent to pursue a science career, and researcher self-efficacy [21–23]. While SIPs intend to enhance diversity and the program components have proven successful for diverse students over the years [23], institutions must institutionalize and sustain effective practices to enhance their efforts to ameliorate the lack of diversity in STEMM fields [24]. Additional research is needed to understand how institutional SIP components are sustained beyond the life of a grant.

### Toward program sustainability and/or institutionalization

In examining program longevity, it is important to define key terms that are often used in program intervention literature. Throughout this paper, we use the terms sustainability and institutionalization. A prior study identified sustainability as “maintaining, and where necessary, evolving or adapting efforts as needed to continue the outcomes of an innovation, and that advocacy from leadership is critical for sustaining innovations” [10 (p.3)]. The authors noted that an intervention might move beyond sustainability and toward institutionalization, which is distinguished as being embedded within the institution over the long term. A sustained activity might utilize external funding to continue, whereas an institutionalized activity is further embedded into the institution’s operations via institutional funding and/or integration into existing units or activities. Additionally, a sustained activity’s ability to maintain itself may seem precarious year after year, whereas an institutionalized activity is secured with leadership support (typically from multiple central leaders in cases of turnover) and structural changes to support the activity’s continuity [12]. These examples highlight how sustainability and institutionalization are similar, yet distinct, and how program activities can move between states of being sustained and/or institutionalized. We examined BUILD program elements that were sustained and/or institutionalized to broaden our understanding of both types of longevity with respect to grant-funded programs, utilizing implementation science to understand the necessary contexts for effective program sustainability and/or institutionalization.

### Framework: Implementation science and institutionalizing change efforts

Initially stemming from the healthcare field [25,26] and adapted into higher education research [27], the field of implementation science seeks to examine how evidence-based interventions are translated into practice [9]. It focuses on understanding “what is actually enacted, how an innovation is enacted, and why the contexts, conditions, characteristics, and other influences shape innovation enactment as they do” [28 (p.172)]. Of the many facets of the BUILD grants, one aim was to implement novel practices, adapted to meet the needs of each institution’s “context.” While there is no one definition of context in implementation science, Gagliardi et al. [29] describe context as a multi-dimensional construct that refers to anything beyond an intervention’s direct implementation and outcome; thus, whether preexisting or developed, context is the circumstances in which any intervention exists regardless of the presence of the intervention itself. Stetler et al. [30] go beyond a neutral understanding of context and further describe the idea of “discouraging or encouraging” circumstances by distinguishing receptive and non-receptive contexts. The authors state, “A receptive context has ‘features... (and also management action) that seem to be favourably associated with forward movement’; and a non-receptive context has ‘a configuration of features which may be associated with blocks on change’” [27 (p.2)]. As described by Stetler et al. [30], specific contextual factors facilitate what interventions are possible and can advance or hinder change and improvement. This impact requires looking at the elements of context and essential dimensions of the change itself – “the what, why, and how” [27 (p.2)]. For our study, we focused on examining contexts that support sustainability and/or institutionalization of previously grant-funded activities and consider the interplay between the grant-funded activity type and contexts that affect an activity’s likelihood of sustainability.

### Examining receptive contexts in higher education

While the organizational change literature discusses factors necessary to create change (processes) and the organizational culture literature has examined norms and values [12,31,32], little literature exists on how the notion of receptive contexts can be applied to sustain programs and practices that support individuals from underrepresented backgrounds in STEMM fields. In fact, Butler [33] and Morrison and Misener [34] are two of the few studies that discuss receptive contexts beyond interventions in the healthcare field. However, there has been one study that focused on the context of structure and support for SIPs that found that legitimacy from departments and the broader institution is important to secure financial sustainability [35]. This study expands on these ideas and the notion of receptive and non-receptive contexts presented by Stetler et al. [30], as adapted from Pettigrew et al. [36]. Pettigrew et al. [36] outlined primary elements of context that shape strategic change in healthcare organizations, including environmental factors, leadership, and organizational elements. Stetler et al. [30] then tested these ideas in looking to institutionalize Evidence-Based Practices (EBP) in other healthcare settings. Through two case studies studying healthcare sites, the authors found that organizations that have contextual features, such as a culture of support for EBP and coherent policy, that are “highly receptive” to a change in the organization and its practices exhibited higher levels of institutionalization of EBP. EBP and contextual features exist across organizations, including in higher education.

Since BUILD was an intervention, similar to the implementation of EBP studied by Stetler et al. [30], we inductively examined the data from BUILD sites to identify elements of receptive contexts for grant-funded student and faculty activities and initiatives that were reported as sustained and/or institutionalized. By understanding contextual elements, we can better understand program sustainability and why different contexts enable higher levels of success in sustaining and/or institutionalizing grant-funded STEMM training activities.

## Methods

The research team drew from qualitative case study data of BUILD programs to examine efforts to institutionalize program elements. Pseudonyms were used throughout the manuscript to anonymize institutions, departments, and participants.

### Multiple case study: Selection and site visits

We used a multiple case study design [37] that enabled us to identify programmatic elements that were sustained and/or institutionalized across the BUILD institutions and to examine similarities and differences across each of the sites. This was the ideal design for the study as the grant was designed to encourage novel approaches across universities. Each university context also informed the way the program was implemented. Simultaneously, looking at each university as a case, we were able to draw lessons that would apply more broadly to other universities.

The study began January 1, 2022, and data collection ended on December 31, 2022. We began by examining each site individually. The research team conducted virtual and in-person interviews ranging from 45 to 90 minutes with a total of 240 participants (see Table 1), including principal investigators, BUILD program directors and staff, faculty participants, program partners, and university leadership (i.e., presidents, provosts, and deans), at each of the 10 BUILD sites. Virtual interviews were conducted in spring and summer 2022, and in-person interviews were held during one- to two-day site visits in fall 2022. While interview protocols focused on the same research questions, questions were adjusted based on the interviewees’ relationship with BUILD. For example, BUILD staff were asked to describe their experiences with BUILD while administrators were asked to share evidence about BUILD’s impact on the campus more broadly. This allowed for a better understanding of each institution’s progress toward institutionalization of the BUILD activities. Interviews were recorded, transcribed, and uploaded into Dedoose software for qualitative analysis.

**Table 1:** BUILD sites and institutional characteristics.

Institution	Institutional Type and Minority Serving Institution Status	Institutional Characteristics	Participants (Faculty, Staff, and Senior Administrators) (n = 240)
**University A**	Public HSI/AANAPISI	Undergraduate Enrollment: 35,000+ Admission Rate: 25–50% Pell Grant recipients: 54%	28
**University B**	Public HSI/AANAPISI	Undergraduate Enrollment: 25,000–35,000 Admission Rate: 25–50% Pell Grant recipients: 51%	25
**University C**	Private HBCU	Undergraduate Enrollment: < 5000 Admission Rate: 50–75% Pell Grant recipients: 54%	29
**University D**	Public HSI	Undergraduate Enrollment: 15,000–25,000 Admission Rate: 100% Pell Grant recipients: 57%	25
**University E**	Public Emerging HSI	Undergraduate Enrollment: 15,000–25,000 Admission Rate: 75–100% Pell Grant recipients: 40%	31
**University F**	Public HBCU	Undergraduate Enrollment: 5,000–15,000 Admission Rate: 50–75% Pell Grant recipients: 56%	17
**University G**	Public AANAPISI	Undergraduate Enrollment: 5,000–15,000 Admission Rate: 50–75% Pell Grant recipients: 28%	18
**University H**	Public HSI & AANAPISI	Undergraduate Enrollment: 25,000–35,000 Admission Rate: 50–75% Pell Grant recipients: 45%	33
**University I & Research-intensive partner institution: University K**	Private Catholic N/A	Undergraduate Enrollment: 5,000–15,000 Admission Rate: 75–100% Pell Grant Recipients: 29%	20
	Public N/A	Undergraduate Enrollment: 15,000–25,000 Admission Rate: 75–100% Pell Grant recipients: 45%	
**University J**	Public ANNH	Undergraduate Enrollment: 5,000–15,000 Admission Rate: 50–75% Pell Grant recipients: 21%	14

*Note:* AANAPISI = Asian American Native American Pacific Islander-Serving Institution, ANNH = Alaska Native/Native Hawaiian-Serving Institution, HBCU = Historically Black College and University, HSI = Hispanic Serving Institution

### Coding and cross-case analysis

After data collection, the team took a multistep approach to coding that included deductive and inductive coding. The initial codebook was developed deductively based on the research questions and the hallmarks identified by the larger consortium to categorize data into institutional-, faculty-, student-, and program-level categories. After site visits, the research team met regularly to further refine codes and definitions. Last, the research team completed a series of coding tests for each code level (e.g., institutional level, faculty level) in Dedoose to reach interrater reliability, calculated in Dedoose using a Pooled Cohen’s Kappa coefficient [38]. After conversations refining code definitions, all five coders reached a score above.80, which was sufficient to proceed with independent coding [39].

For this study, the research team also conducted a detailed review of debrief reports and case narrative reports to determine student and faculty initiatives that were sustained and institutionalized. Following each site visit, the research team wrote debrief reports of the visits and case narrative reports to summarize the activities at each site. Utilizing these reports, we conducted a within-site analysis by creating matrices to visualize and categorize initiatives institutionalized at each of the BUILD sites [40]. We also examined a subset of codes related to institutionalization and sustainability (see Table 2) to confirm and finalize an inventory of institutionalized activities through the use of a summary table [41].

**Table 2:** Institutional level codes from case study Phase II codebook.

*First Cycle Coding: Descriptive*	
Code	Institutionalization and/or Sustainability
**Subcodes**	• Finances • Research and Research Infra/structure • Tenure and Promotion (policies) • Diversity Training and/or Faculty-focused Training Efforts • Student Advising and/or Student Training/Development Efforts (structures, processes, programs, workshops) • Physical Structure(s) • Staff/ing • Intra- and Inter-Institution Partnerships • Curriculum/Courses • New Elements • Amplifying Existing Elements • Challenges to Institutionalization and/or Sustainability • Lessons/Suggestions for Institutionalization or Sustainability
**Code**	Institutional Culture/Climate
**Subcodes**	• Context • Leadership (top-down climate)
*Second Cycle Coding: Pattern and Thematic*	
**Code**	Structures
**Subcodes**	• Preexisting Offices • Physical Infrastructure • Inter-institutional Partnerships • Intra-institutional Partnerships
**Code**	• Personnel
**Subcodes**	• Leadership Commitment • Leadership Turnover • Faculty Commitment • Willingness to Commit Resources
**Code**	• Fiscal Conditions
**Subcode**	• Financial Status of Institution • Ability to Obtain External Funding
**Code**	• Mission Alignment
**Subcodes**	• Mission of Colleges • Strategic Plans • Receptivity to Data Use

We then used a subset of the coded excerpts focused on the institutionalization of student and faculty training initiatives to understand contextual relationships. We conducted an additional round of open coding [42] to determine the contexts and conditions that influenced institutionalization efforts. Next, we conducted a second cycle of coding informed by the theory on receptive contexts to determine relationships within the data set. Specifically, we used pattern and thematic coding [42] to categorize the data and identify emerging themes. We then developed matrices to engage in cross-case analysis and identify similarities and differences in the contexts and conditions that influenced institutionalization efforts across sites, following the priorities of multiple case study [37].

### Ethics statement

All participants provided verbal consent. The UCLA Institutional Review Board approved this study.

### Trustworthiness

Triangulating our findings across various forms of data informed our understanding of which elements of the BUILD program were sustained and institutionalized. At the end of each site visit, the research team met with BUILD program leaders to discuss emerging findings related to institutionalization and impact of the program. During the meeting, program leaders had an opportunity to clarify observations and offer any additional information about the BUILD program. Each site was also provided with a debrief report summarizing key findings and observations. Program leaders were asked to provide feedback to ensure accuracy of the information. The research team wrote case narrative reports that were reviewed by multiple team members to provide additional institution-specific context [37]. Throughout the process, members of the team wrote analytic memos and notes to add context and clarity to observations. We presented initial findings to the other members of the CEC for additional feedback to ensure accuracy of our interpretation of the findings. Multiple team members participated in each step of the process to triangulate findings within and across sites [37].

### Positionality

The authors acknowledge that their shared history informed their approach to this study. All authors are invested in identifying approaches to institutional change that promote STEMM equity in higher education. Four co-authors have research backgrounds in organizational theory, and the first author has a background in program evaluation. All authors study STEMM intervention programs. Together, these fields of expertise shaped our conceptualization and analytic lens of the study. The larger evaluation of BUILD was enacted using a culturally responsive evaluation framework [43]. As members of the CEC and outsiders to the institutions, we were able to examine similarities and differences across all BUILD sites. Additionally, of the co-authors, all identify as Latine and/or multiracial, four identify as first-generation college students, one earned a bachelor’s degree in mathematics, and another earned a master’s degree in molecular biology, mirroring many of the student and faculty participants’ identities. As a result, the co-authors were able to bring an understanding of what it means to navigate institutions from a marginalized positionality and value diversity in education and training environments.

### Limitations

There are several limitations of our study. First, we were limited in being able to name the sites to protect the anonymity of institutions and individuals involved in the study. While this limited the extent to which we were able to provide in-depth descriptions and contextual details for sites, we tried to provide general institutional context details where possible. While most of the findings report the most salient elements across sites, we do include some site-specific findings. Additionally, we provided institutional details for each site. Lastly, we collected data two years prior to the end of the 10 years of grant funding. While efforts were made to confirm what was fully implemented and institutionalized, not all program elements could be confirmed. Similarly, while institutionalization often occurs past the life of a grant, this paper is able to discuss several elements that were being institutionalized throughout the grant and initiatives that sites were taking to sustain the work beyond extramural funding.

## Findings

To identify the BUILD initiatives that will be sustained or institutionalized after funding ends, we developed an analytical inventory of BUILD-funded student- and faculty-level program initiatives that sites were moving toward or had already sustained and/or institutionalized (Table 3). We found that the most common areas sustained included undergraduate and graduate programming; curriculum and courses; faculty development mentor and research training; and partnerships with collaborating institutions.

**Table 3:** Research training activities expected to continue after BUILD funding ends.

Institution	Sustained Activities: Student	Sustained Activities: Faculty	Sustained Infrastructure: Institution
**University A**	Transfer student support program and center Undergraduate Research Office Summer research program Work study program for students to do research BUILD-created courses	Community-oriented health equity research center (Plans for) Faculty training Seed grants for faculty	Health equity center building space and BUILD Library space Community partnerships
**University B**	20 BUILD student scholarships per year Learning communities Intensive mentoring Undergraduate Research Opportunity Program A week highlighting research activities on campus Research Certificate Program BUILD courses expanded Federal Work Study for undergraduate research Graduate student training program	Faculty training in Faculty Development Center Faculty Search Equity Advocates	BUILD Center and Offices in Student Center Research lab renovation Active learning classroom renovations Community College course sequence Faculty training across campuses in state-wide system Interdisciplinary partnerships between colleges MOUs to sustain BUILD
**University C**	Student training workshops Tutoring and supplemental instruction STEM Advisor Undergraduate research shadowing Summer Research Symposium Undergraduate research experiences in courses	Faculty training to prepare mentors and advisors	Lab equipment Partnership with other institutions Tracking/monitoring data
**University D**	18 BUILD scholars funded per year Student wellness programming Annual Research Symposium Summer Research Opportunities with Partners Research driven courses	Grant writing training Mentor training Faculty director role	Classroom renovation Lab equipment High School partnerships R1 partnerships Community College partnership Evaluation tracking
**University E**	Student enrichment sessions Peer mentoring Research learning communities Summer Research Academy Research Courses Transfer pathway for students from a partner institution with a large Pacific Islander student population	Mentorship training with R1 University	Hub for all SIPs on campus Partnership with R1 Partnerships with Community Colleges
**University F**	Entrepreneurial student training module in Office of Undergraduate Research Learning community space Mentoring and peer mentoring Professional/Career Development events Undergraduate Research Course	Institutional support for trainings, professional development, and grant management Mentor training Faculty pilot grants Course-releases	Upgraded research facility Student research space Lab equipment Partnership with R1 Replication of programs at other campuses; hope to form a consortium
**University G**	Student training opportunities Student success coach Living Learning Community Alumni mentoring STEM resource hub Summer program (maintained via fees) STEM writing and ethics courses	Faculty training certification Faculty training considered in tenure and promotion practice	Renovated learning space
**University H & Research- intensive partner institution: University L**	NIH diversity supplements Training modules for students Post-baccalaureate programs Supplemental Instruction Program Peer mentoring Research-oriented curricular changes Courses: Health equity, Graduate TA pedagogy, and Research with communities	Ongoing faculty training Annual writing retreats	Expansion of furniture and office spaces Partnership with R1 Additional university grants Partnership for ongoing training with outside institutes Leadership placed in administrative positions
**University I**	Summer research Graduate student fellowships Coach for student success Research concentration Undergraduate Research Office in a College Research-oriented courses	Faculty initiatives in existing centers Faculty Search Advocates Mentorship considered in tenure and promotion requirements Hiring ads revised through cultural competence lens	BUILD student space Renovated lab Articulation Agreements with R1 and local Community College Search Advocate training
**Research- intensive partner institution of University I: University K**	Continue scholar program Formalized MOU with administration Student opportunities in the school of medicine Post-baccalaureate program Partnership with another undergraduate student program Research-oriented courses		Renovated BUILD classroom
**University J**	Undergraduate research experiences MA program focused on program philosophy Student Success Center Student advising and mentoring access Research-oriented course sequence	Curriculum Development Week Mentorship included in Tenure and Promotion evaluations	New furniture and technology in classrooms High school community partnerships Community College partnerships

### Student-level university elements most likely to be sustained

Many of the sites prioritized sustaining the BUILD-funded initiatives that supported undergraduate students. Some BUILD team members shared that it was easiest to “make the case” for maintaining successful programs that support student retention, graduation, and other indicators of academic progress. We summarize the main student-focused activities most likely to be sustained post-grant.

#### Undergraduate scholarships, research, and biomedical training.

All BUILD sites developed a student training program, which provided the most intense exposure to BUILD activities since it required students to engage in structured programming, including a combination of professional development activities, mentorship, and undergraduate research opportunities. While programming varied by site, all programs included financial support in the form of scholarships and stipends. All sites expressed a desire to continue scholar programs but were faced with challenges in finding substantial funding to continue all elements of their programs. Most sites received institutional funding commitments or obtained external grants to continue some initiatives. Three sites will continue to have scholar programs with cohorts each year that are funded by the institution. (We discuss how the context of these sites played a role further in the findings.) Three other sites are sustaining downsized versions of their scholar programs through external grant funding. Another site reported plans to partner with the Honors College as a way of obtaining funding for students selected as scholars.

Each BUILD site selected the approach that best fit its local conditions. This resulted in different trajectories for expanding undergraduate research opportunities, particularly to enhance participation of students from groups underrepresented in biomedical fields. For example, at University J, a formal staff position developed as part of its BUILD program that provides students with consistent access to support as they navigate their STEMM majors and undergraduate research training experiences. Other sites diffused initiatives into the larger campus community. At some sites, this effort was scaled-up by establishing campus-wide undergraduate research offices due to conditions that facilitated this approach. At other sites, the evidence of successful summer research programs led to institutions absorbing the program. For example, University G and University A will be continuing their summer programs by integrating with a larger summer program at the university or facilitating the program through their undergraduate research office, respectively. We will discuss the conditions that shaped these varied outcomes in a subsequent section.

#### Post-baccalaureate programs.

In addition to undergraduate access to research, three BUILD sites will continue to offer post-baccalaureate opportunities. At University C, post-baccalaureate technician positions are being sustained on a smaller scale through faculty writing these positions into grants. Similarly, University L offers a post-baccalaureate program where students are clinical research coordinators. Lastly, University K has submitted proposals to continue an existing post-baccalaureate program aligned with BUILD. All three of these institutions are able to continue these programs because they have faculty members committed to maintaining them. These programs are continuing to give students opportunities beyond the scope of the grant to support long-term persistence in STEMM fields.

#### Curriculum and courses.

All BUILD sites will be maintaining some changes to curriculum and/or courses. As stated by an administrator, “In higher ed[ucation], if you can do anything that’s part of the curriculum, then you have a chance of sustaining that and their structures and resources and things around that, that you can build off of.” All BUILD sites created new research-oriented courses through BUILD, and two sites added research elements into previously existing courses. Many of the courses are Course-based Undergraduate Research Experiences (CUREs), which span several disciplines and foci within the biomedical field. Several sites are increasing access to research by scaling up CUREs for all students enrolled in introductory STEMM courses.

Additionally, some sites have developed new programs for students to be able to receive formal access to research. University J’s program utilizes a unique philosophy that integrates the health of animals, the environment, and humans. This is now not only in the undergraduate curriculum but is also the foundation for a newly developed master’s program. Additionally, University C created a research minor, and University I has created a research training certificate for students.

### Faculty-level university elements most likely to be sustained

BUILD sites implemented initiatives that were directed toward faculty, primarily through activities such as mentor training, pilot research and equipment grants, course buyouts, and changes in faculty hiring and tenure and promotion policies. While many of the BUILD-funded activities have terminated at the end of the funding period, some sites are focusing their attention on institutionalizing training and hiring practices campus-wide.

#### Faculty training.

Faculty training largely focused on improving mentoring and teaching, and supporting faculty grant-writing. While practices that faculty have learned thus far in training will continue to impact the universities, several sites were working to institutionalize these programs. At one site, an academic administrator noted that “the training that they did for their faculty or staff, the support services that they provided to the students, really built on some of the things that we were already doing” and that they think inviting faculty across the university to development opportunities “will continue.” They will likely continue because training was effective and aligned with the goals of the university.

Several campuses focused on creating training that was scalable both within and beyond the institution. At University G, the faculty development certificate program trains faculty to adopt teaching practices to support diverse STEMM students. While this began with a cohort of faculty primarily in the sciences, it has now expanded across disciplines, and operations will continue to be maintained by the Faculty Teaching Center with colleges contributing to the cost of running the initiative. One evaluator mentioned, “[the faculty training program has had] an institutional impact outside of STEM, to other areas as well. So that’s a real success story that’s going to continue.” University F also scaled its faculty pedagogy training campus-wide as it reflected the greater goal of the university. As one PI mentioned,

Our now-Provost, former Dean in our school, came in and we explained [active learning pedagogy training] to him. Instead of struggling to find a way to do it, he immediately embraced it, and said, ‘this is great. I want all my new incoming faculty to take this and I’m going to encourage everybody to take it.’ And now it is institutionalized in a new office.

This work at both sites reflects an effort and success at scaling the faculty programming more widely. In this process, the program was able to be institutionalized as it was actively integrated into different parts of the institution.

Further, some BUILD sites are spreading their training to other universities. Three of the BUILD institutions created their own consortium within BUILD because of shared institutional characteristics and structures. The BUILD-funded campuses are leveraging their connection with one another. University B developed six mentor training modules that are now made available across this three-site consortium. University A has expanded some of its faculty training beyond its campus by creating faculty mentor training models using a Critical Race Theory framework. Faculty training was also expanded for graduate student teaching assistants. At University L, this effort emerged from faculty initiatives, with the basic content of the training institutionalized into a pedagogy course:

The timeline [began with] the [program’s] readings, which then informed a workshop that several of us gave to tenure track lecturers, faculty, and graduate teaching assistants … and that workshop was funded by BUILD. And then that led to the pedagogy course [which] uses some of those readings that we did in the [program workshop]. That’s now, the graduate teaching assistant pedagogy course [that] is now institutionalized.

Other initiatives lack the same amount of support across universities. Most sites are discontinuing their grant-writing training and faculty writing retreats as independent programs, but several institutions have embedded some aspects of support for grant writing. For example, University A institutionalized the training through the office of the Associate Vice Provost of Research; University F is continuing to give course releases for faculty to focus on grant writing; and University D is integrating grant-writing training into other faculty initiatives. Additionally, sites had invested in internal grants and purchasing equipment for research. While most sites are discontinuing BUILD-funded pilot grants, University F is continuing to offer pilot grants to faculty funded by the institution.

#### Changing faculty hiring practices.

Several sites have also institutionalized BUILD-initiated changes to faculty hiring practices as they are a way of creating change that can be easily maintained without ongoing funding. Two sites institutionalized “faculty search advocates,” members of a faculty search committee who receive training to ensure that the hiring is fair at all stages of the process. While the original grant aims focused on biomedical fields, faculty search advocates became part of every department search process at these two sites. At University I, one core director highlighted how this change is necessary and reflected on campus:

It’s a campus-wide policy that you have a search advocate on faculty and administrative search committees to ensure that there is equity in the process... because the search process is a racialized process. That’s one of the systemic things we’re trying to undo. And so by having a search advocate that’s checking for bias and checking for, “Well, why are you disqualifying this candidate? I see that they have eight publications,” or whatever it is, that person is there to check you and make sure that you’re trying to weigh candidates not the same way, but equitably.

At other campuses, policies were oriented toward their differing campus needs. At University B, their version is fully paid for by the institution and added to the budget. Thus, each campus adapted the work to their own context.

### Institutional level elements most likely to be sustained: Infrastructure developments

Although infrastructure is a broad concept, we use *infrastructure* to refer specifically to the forms of physical, digital, and organizational infrastructure that BUILD supported to “strengthen institutional research training infrastructure and capacity” and to develop “infrastructure to support data collection to evaluate the impact and effectiveness of the proposed program,” as described in the original RFA.

Several sites invested in research infrastructure, both physical and digital, that now appear to be sustained beyond the grant period. Several sites have built up their initiatives to support diverse populations of students in biomedical research by creating offices of undergraduate research. At many of the sites, offices have been institutionalized and extend the services beyond students directly involved in BUILD. At one site, the office of undergraduate research has not yet been formalized but exists within a STEMM-focused college. This illustrates how research infrastructure can occur within receptive departments or colleges, even when not prioritized institution-wide.

At some campuses, the diffusion of knowledge regarding the effects of research training enhanced existing research infrastructures. For example, University E previously enacted several scattered undergraduate research programs. BUILD enabled these to be integrated into a single institutional space dedicated to student engagement in research.

[The Office of Undergraduate Research] has supported that [equity] effort. …It started because they’re renovating one of our major science buildings, … centering it on making it a space that feels inclusive,...intentionally planned to be a place where students from diverse backgrounds feel at home.

The BUILD team used grant funding to renovate or build physical infrastructure to support undergraduate research. Establishing these hubs for undergraduate research, or renovating laboratory spaces, would not have been possible without BUILD’s investment to enhance research capacity. These spaces will continue to serve students across several STEMM training programs. Similarly, University D created a physical space in a renovated building where undergraduate research programs are housed along with student success initiatives.

We also saw evidence of program evaluation elements becoming institutionalized. Each BUILD site was required to have a concentrated focus on evaluating their respective program. Evaluation methods and tracking of students and alumni developed for BUILD will remain part of the institution. For example, evaluation systems at University C and University D are both being maintained and diffused into central offices that focus on monitoring and evaluation. These examples from the BUILD sites of sustaining and institutionalizing efforts are significant because they demonstrate program longevity beyond funding.

### Receptive contexts for sustaining grant-funded activities

We identified four elements of institutional contexts that aid or inhibit institutionalization: institutional financial status, structural conditions, commitment of administrators and faculty, and institutional priorities. Contextual elements are receptive if they provide a condition that makes change more likely; in this case, if this context would aid institutionalization. Thus, some contextual elements became more receptive over the course of the grant-funded period, in part due to BUILD team leadership contributing to cultural and structural shifts on campus. Every institution varied regarding the level of receptivity within each of the four contexts; however, how different contextual elements work together can add to the understanding of why elements were or were not institutionalized.

#### Contextual element 1: Institutional financial status.

Institutional financial status refers to the availability of funds within the university and, consequently, the availability of funds for interventions. Across all sites and programs, financial viability emerged as the largest factor that affected programs’ ability to sustain program activities. Sites sought funding from institutional budgets, private donors, and/or external grants to maintain interventions. Thus, while some sites were able to be successful primarily through these outside sources of funding, having fewer institutional funds available limited the extent to which the institution was receptive to sustaining BUILD activities.

While all sites expressed the difficulty of having significantly decreased funding after the end of the grant, this was further exacerbated at institutions that were experiencing financial challenges. For example, leaders at University H, University B, University J, and University E all mentioned that they were facing declining funding. As one administrator described their financial status:

[Institutionalizing faculty initiatives has] been a challenge as we’ve been cutting a lot of money from our budget. What was, when I started here, an $11 million dollar shortfall, is now turning into a $20 million dollar shortfall.

This speaks to the drastic changes that the university is experiencing from declining enrollment and the long-term effects of COVID-19. The challenges resulted in senior administrators seeking to cut programs geared to a select number of high-achieving students with more resources, limiting what aspects of BUILD can be maintained. University J was another campus with an extreme funding deficit, having cuts of 25% and 11% within 3 years. One leader mentioned,

[Institutionalizing a BUILD-created staff position] right now, is not in the cards. Again, the reduction in support for the university was significant over the last three years. That really, I think, has hampered a lot of our ability, despite great commitment to the cause to actually provide the support that we would really love to support. Because, are you going to fire faculty in order to hire a [staff member] or two?

Regardless of university leaders’ desire to continue BUILD activities, they could not financially support BUILD because the institution was struggling to fund faculty and other pre-existing initiatives. Consequently, substantial financial shortfalls that colleges and universities face created a context that was not receptive to sustainable change, despite evidence of being both innovative and successful.

Some sites developed a more receptive context by finding additional means of funding and resources needed to sustain programs. The condition of limited institutional funds led some sites to seek funding from outside of the institution. Many program directors applied for additional grants, such as the NIH U-RISE grant, to maintain student training programs; however, not all sites were equally successful at obtaining external funding and programs that did receive funding were informed of cuts in 2025 [6]. Other sites developed ways for the institution to redirect campus income toward continuing undergraduate research. For example, University B captured grant indirect costs that will enable 10 scholars per year to be funded for another 10 years, along with an additional 10 scholars funded by the provost’s office. Similarly, University J has a model where 1% of all indirect costs from grants are used to support undergraduate research. These alternative models help address the financial burdens institutions face.

#### Contextual element 2: Organizational infrastructure and partnerships.

Organizational infrastructure consists of inter-institutional partnerships, intra-institutional partnerships, and physical and digital infrastructure, which are integral to facilitating program sustainability, because they enable shared resources. Additionally, preexisting offices and infrastructure provide a readily receptive context for sustainability, and some sites’ grant activities facilitated the expansion or creation of new offices/centers that subsequently sustained additional grant activities.

Strong inter-institutional partnerships increased the likelihood that BUILD sites sustained elements of their training programs, mainly by sharing resources. For example, Universities A, B, and H formed a smaller within-DPC consortium to incorporate lessons from grant implementation to scale up support for students and faculty. One PI explained how the partnership developed:

The three BUILD programs within the [consortium], we’re uniquely positioned. Of the ten BUILD programs, three of them are within the [state-wide system], I said, “We serve the same students! So why don’t we work together and see if we can pool our resources, and develop a synergy to work with our students and faculty?”

The PI went on to explain the synergy developed by lessons learned among the three sites to improve program implementation, effectiveness toward target populations, and program sustainability. Their connection to the system’s administrative office enabled a combined effort to institutionalize doctoral student mentorship and training. This partnership between sites A, B, and H required significantly less financial support provided by a single institution. Thus, these connections facilitated institutionalization of training elements despite declining institutional funding.

Strong partnerships with research institutions also facilitated program sustainability, particularly for summer undergraduate research experiences. For example, one BUILD team member at University C shared that partner institutions have committed to hosting students to do research over the summer without requiring funding from University C. Maintaining these inter-institutional partnerships reduced the costs for BUILD sites that initially used their grant budgets to fund students’ summer research experiences and internships and/or to add research institutions on as sub-awardees. While many student training interventions are expensive to sustain, research partner institutions can share or cover the cost of student summer experiences.

Intra-institutional partnerships were key elements of a receptive context for program sustainability. Nine of the 10 sites utilized pre-existing offices and centers on campus to aid in institutionalizing different aspects of the program. At some sites, BUILD-funded program activities were integrated into these offices and centers, consolidating costs for staff, materials, space, and other resources to run the activity. For BUILD-developed student programming, sites primarily worked with student success centers, undergraduate research offices, and/or specific colleges to implement a transition plan in advance of the end of NIH DPC funding. Preexisting offices not only provided a place for the grant-developed activities to continue but also unlocked staff time and funding from those offices to devote to continuing BUILD initiatives.

The existence of additional STEMM training programs on campus enabled opportunities to organize and coordinate programs to streamline students’ pathways into STEMM training activities. University E leveraged the ability to connect with other science training programs on campus. One program manager explained the logic of their efforts:

The idea is take the community, get them to the [summer research academy], get them into [BUILD], and then kind of funnel them into LSAMP, McNair, U-RISE, the president’s funds for DACA students. So, it’s just kind of a way to help them explore.

This synergizing underscored that all the undergraduate research centers had a desire to work toward longer-term undergraduate research support. Thus, having a variety of STEMM-related training efforts on campus that intentionally coordinate and synergize resources and efforts contributes to a context that is receptive to institutionalizing grant-funded activities.

Some campuses were unique in building a receptive context through non-academic partnerships. For example, University G sustained their BUILD-developed STEMM Living Learning Community (LLC) through an endowment gift by partnering with their institution’s advancement office and residential life office:

We have a donor through the university that gave a million-dollar endowment for the LLC. We work[ed] with our [advancement office] and that’s how we got that partnership. Recruitment [for the LLC] will obviously look a little bit different, because I’ll be working directly with [the residence life office] just to fill all those spots as opposed to working with our evaluation team and our internal BUILD team, it will just shift.

The context of strong partnerships with a successful advancement office that was able to obtain financial support, in combination with a student affairs-related office that provided the infrastructure for delivering STEMM training opportunities to students, helped facilitate longevity of this grant-funded initiative. Since students are paying for housing already, the endowed funds can stretch further to maintain the LLC.

To sustain faculty-level activities such as training in mentoring and grant writing, several sites utilized an existing faculty training center and/or office on campus to manage and facilitate the activity developed during the grant-funded period.

Physical and digital infrastructure is another important condition for institutionalization. Several sites leveraged preexisting infrastructure to maintain some BUILD-developed activities. University A has received both university and grant funding to “transform some of the training to online training so that students could [participate in trainings].” Along with University C, they were able to leverage existing websites so that training sessions can be delivered online at little to no ongoing cost. Further, the investment in software during the BUILD grant enhanced the institution’s digital infrastructure:

We also use some carryover funds to help the university purchase some key large software packages that we will continue. …You can do appointments, like now all of our resource centers, tutoring centers, are using it for appointments for students to be tutored, like we did total drop-in tutoring, pre-COVID. … And obviously, we’re continuing the maintenance on those systems. So not on BUILD’s dime [in the future].

University C’s use of this software enhanced their preexisting support for students. As this software was already transitioned off BUILD funding, the condition of having this online system aided in being able to enhance the resource centers’ ability to reach students. As well, University E’s partner community college utilized their learning management system to maintain a repository for students to learn about announcements and events. Maintaining this repository was described as a “low-stakes” activity because it can easily be sustained.

#### Contextual element 3: Commitment from central administration and STEMM faculty.

Having the commitment of central administration and STEMM faculty at the institution is critical to successfully sustaining initiatives. As the actors in the university with the most authority, the response from deans, provosts, and presidents either aided or inhibited the continuation of grant-funded activities. At University A, one of the PIs mentioned, “What I learned there is if you have direct access to the provost, which my partner did, and the provost sent out emails to people, we got droves of people involved, including her.” This speaks to the central administrators’ capacity to influence which grant-funded initiatives gain traction throughout the university.

Substantial institutional commitment from their central administrations for BUILD’s sustainability was key to a receptive context. For example, university leadership expressed how they would actively work toward further funding these initiatives. At University B, a central administrator noted that in addition to the already committed funds, they saw the BUILD grant as an opportunity to expand the institutionalization of the larger research infrastructure of the university. Their commitment to the overall programming can contribute to future funding and, consequently, further program sustainability. As well, an administrator at University G expressed a similar willingness to absorb the cost of faculty training and a dean at University I committed to “picking up more and more of the salary” for the student success coach.

Active work and direct investment in the BUILD interventions support program sustainability. Alternatively, the lack of leadership commitment can reduce the likelihood of institutionalization. For example, one site stopped providing substantial internal grants as the campus leadership did not see sufficient impact. As well, this lack of commitment can be due to broader university conditions such as central leadership turnover. As one core director mentioned,

I think one [challenge] is that there’s a constantly changing environment in terms of people, the people that we have to work with. So, we’re on our third or fourth president now, our fourth provost, our third VP for research. So that transition in leadership has been, I think, something that has made it difficult to, again, really take full advantage of everything that university could have done with BUILD.

At this site, the continuous changes in leadership inhibited the scale at which BUILD could be sustained. However, while turnover created a barrier to program sustainability, it did not halt it completely as deans and/or other campus leaders worked to sustain momentum toward institutionalizing grant activities.

Faculty commitment to enhancing diversity in STEMM fields via teaching and mentoring is essential. Having a faculty culture that values and works to improve their capacity to support STEMM students is a condition that facilitates institutionalization of student programs. It is particularly important when grant funding ends because incentives such as stipends may no longer be provided to faculty for their participation in mentorship-related training and activities. As one core director noted, “you rely a lot on faculty,” particularly when it comes to mentoring and advising students. For example, several faculty members demonstrated a desire to continue mentoring students after participating in BUILD activities. At University H, one faculty member noted that “faculty members are really eager to mentor [BUILD] scholars,” indicating that faculty value developing relationships with students. Faculty at some sites showed a willingness to voluntarily mentor and participate in training without financial or other incentives. One faculty member at University A discussed her commitment when she said, “I wish there would be some funding to be able to continue to support some of that, but even without it, I [am] committed to these students.” This faculty member felt a responsibility to students and a willingness to continue mentoring, a sentiment also expressed by faculty at University B and University C.

#### Contextual element 4: Alignment with institutional priorities.

The overall priorities of the institution, noted in its mission and strategic plan, are central to grant-funded program longevity because university resources are allocated toward institutional priorities. Thus, a grant’s overlap with current institutional priorities supports program sustainability.

A key component of receptivity was the extent to which the aims of BUILD were reflected in the university’s larger strategic plan. When talking about the difficulties of a limited budget, a provost commented:

This is also a really important thing, and I think it’s particularly important for people who are working on these types of projects that don’t have a provost, or a dean involved, and that [are in] alignment with this. The way to obtain funds for an initiative, or whatever you want to do, is you have to align it with the strategic priorities of the university. And I think that’s, to a certain extent, what [another administrator] and I can do. We know what the strategic plan is and [BUILD is] perfectly aligned with what we’re doing.

The administrator’s comment is framed as a reflection of not only personal experiences but also what lessons those experiences may offer to other universities. As a provost, that individual found that alignment with strategic priorities of the university is an essential element to program longevity. At University B, an administrator also echoed support for BUILD through the way it aligned with their newly launched strategic plan:

We launched a new strategic plan. … The two action zones that I like to try to connect with BUILD are the first one that aims to develop an equitable campus for every individual on our campus, irrespective of their roles. The third action zone is re-imagining faculty. How can [we examine], for instance, our campus retention, tenure, and promotion policies to recognize the hidden work that some of our very diverse faculty members find themselves involved in, particularly service? Very often when they are in an environment like ours, your diverse faculty members are the first mentors of sorts that your marginalized students will reach out to.

University B largely institutionalized their faculty training initiatives, particularly by integrating them into their faculty development center. This strategy reflects one of the “action zones” discussed by the provost.

Being student-centered was one of the highest priorities reported by almost all BUILD sites, considering several of the sites are also teaching-centered institutions. We found variation in the extent to which student versus faculty elements were institutionalized. Student programs were able to draw in more financial support as they could show data on their effectiveness and their alignment with the student-centered missions of the institutions. While faculty training programs were also maintained across most sites, all sites maintained elements of their student-centered initiatives. In part, this is due to student investment showing the most direct outcomes when there is limited funding. One administrator stated that “when you have to cut that much money, it’s hard to, and every penny I get, I try to put towards things that are directly tied to student outcomes.” Further, the same administrator noted that the program had shown “real evidence of what works in getting students engaged in really high-end research and in changing their trajectories.” While student interventions show a direct impact, it can still be difficult to maintain programs when there is a lack of funding. However, as a result of that priority, student interventions appeared to be institutionalized more consistently across BUILD sites.

Central administrators were committed to supporting the continuation of student-focused BUILD activities that aligned with other goals for supporting students. For example, a member of the BUILD team at University J noted, “the administration there is very much interested in engaging and supporting and helping Indigenous students be successful. And so, they’re trying to institutionalize a lot of the mentorship stuff that we’ve done and a lot of the scholarship stuff.” At the time, the institution was focused on enacting key elements of its institutional plan, which aligned with BUILD-developed activities that aimed to support retention of undergraduate students from underrepresented backgrounds.

Some BUILD institutions were able to institutionalize their research initiatives as the schools in their entirety were shifting toward becoming research-focused institutions. Over the course of the BUILD grant, one institution became an R1 institution, and two others were working toward becoming an R1. For example, University F has a distinct goal set by the university: “Overall, it is part of the bigger picture and the bigger goals of [University F] … to go from a purely training institute, a teaching institute, to a research institute.” During the grant, they became an R2 institution, and this movement toward research can be seen in what is institutionalized, such as faculty receiving course release time to focus on research. Additionally, University D became an R1 institution in 2018, and the president and state coordinating board have specified that “doing undergraduate research” is a priority. As a result, offices devoted to research for both students and faculty were accessible to absorb pieces of student training. According to the Office of Research and Sponsored Programs, they also have a surplus in grant money for students from a state funding source and many faculty have become successful at getting grants, making it possible for University D to maintain elements of the BUILD program and continue to fund scholars. The centrality of research in institutional mission has enabled BUILD sites to successfully maintain programs that reflect those priorities.

One challenge faced by BUILD sites that primarily focus on teaching is that conducting research may not be feasible in the same way it is at other institutions. As one faculty member explained, central administrators view research as more of a “hobby” that faculty pursue, rather than something that is part of the fabric of the university. This view is reflected in the other demands required of faculty by the university. For example, at many BUILD sites, there is a high course load that can prevent faculty members from devoting as much time to research:

One [challenge] is that we have a really high teaching load. We teach three courses a term. And so asking faculty to do research when they don’t, quote, “have to,” in the same way…And they’re not necessarily given a break from their teaching duties to make that happen is very difficult. You’re asking people to do stuff on top of what they’re already doing.

Along with other institutions, the focus on teaching makes it difficult for faculty to have time to work on research, regardless of their own desire to conduct research.

Further, the desire for data-driven decisions based on priorities within the university highly influenced what was able to be institutionalized. University J, for example, faced declining funding during BUILD implementation. One administrator stated,

What is important is that there is a [budget] process and once you put that process in and you know the request and what it is for, faculty have to still justify why they need it, what is it promoting? How is it adding to our goals, different goals? And they have a set of [questions on] how does it help with our accreditation process? They have to respond to those metrics, but as they rank and rate high, we try to find funding for as many proposals as possible.

This budget issue speaks to the intertwined nature of institutional financial status and institutional mission. The importance of this condition of being mission-aligned is amplified in importance by the financial constraints, demonstrating how different elements of receptive context may be held in tension with one another.

## Discussion

The BUILD initiative was designed with a multifaceted approach to implementing novel and innovative strategies to enhance the diversity of well-trained biomedical research scientists [31]. Across sites, there have been a wide array of initiatives that sites are actively working to maintain through institutionalization. Our case study of the BUILD initiative provides a rich set of data for understanding specific activities that are most likely to endure long term, particularly because each site was able to develop its own tailored set of activities at the student, faculty, and institutional levels. We discuss here lessons learned from what was most commonly sustained and/or institutionalized and implications for the four contexts identified as key to supporting institutionalization.

### Grant-funded activities most commonly sustained

The most frequently sustained initiatives included student and faculty training, and activities that required few resources to maintain. The two most commonly implemented interventions across all sites were undergraduate opportunities to engage with research and faculty training initiatives. Every institution provided direct programming to students, including continuing scholar programs, integrating research into classroom curricula, and undergraduate research offices. While continuing scholar programs were unique to sites that had direct funding from within the institutions, all sites noted that their grant-developed novel biomedical curricula were the easiest to institutionalize after the BUILD grants ended. Curricular changes are important as previous research indicates that infusing research into undergraduate courses has the potential to increase interest in scientific research [44]. Our findings suggest that sites found curricular changes to be successful enough to try to sustain, that they are easier to sustain than other types of interventions, and that there are key characteristics that can support an innovation’s sustainability beyond the grant-funded period.

Faculty initiatives that were most commonly sustained include training opportunities and shifts in faculty hiring processes. BUILD sites were able to gather data and assess how training was received by faculty, mainly via engagement with workshops and self-reported impacts by faculty. Further, hiring and promotion processes became an avenue to make permanent institutional changes that could enhance diversity in the biomedical professoriate.

Finally, infrastructure changes using grant funds (renovating or developing classrooms, labs, or buildings) open up the possibility of sustaining grant-funded activities. These physical spaces also support the possibility of future initiatives. Many sites also purchased lab equipment to expand student and faculty opportunities to conduct biomedical research collaboratively; however, sites with large purchases of equipment had to ensure that faculty and/or lab staff were in place to continue to facilitate reservations and maintenance of equipment.

### Institutional conditions that aid program sustainability

Ultimately, this study led us to identify four institutional conditions that play a role in facilitating sustainability after grant funding ends. This provides a framework for conceptualizing the elements of context in higher education institutions that aid or detract from receptivity of STEMM education initiatives. Grant-funded activities were sustained when one or more favorable or receptive conditions were present: institutional financial status; organizational infrastructure and partnerships; commitment from central administration and STEMM faculty; and alignment with institutional priorities. Sites either had contexts receptive to those interventions or developed contextual elements to be receptive to the sustained elements. While there may have been elements of context that were not receptive, overall, the context required at least one key element to be receptive to enable the intervention to be implemented and maintained after the NIH DPC funding ended.

### Implications

There are several implications for research and practice. First, no intervention can be maintained without being financially viable. While some interventions require little to no ongoing financial support, most require at least some investment from either the university or external funding sources. Particularly after COVID-19, many institutions lacked the financial resources to supply ongoing funding. Thus, programs were able to compensate for the lack of institutional funds by seeking outside funding, a trend that has become more prevalent in efforts to sustain SIPs [35]. However, those outside sources of funding rarely fully compensated for what the university could not supply. Further, several STEMM training grants that serve diverse researchers and students have been cut by the Trump administration with letters to program directors stating “This award no longer effectuates agency priorities” [6]. As a result, the necessity of an institutional funding commitment is more important than ever.

Next, in order for funding to be utilized, personnel are needed who actively work to implement the sustained interventions. A key element of contextual receptiveness to an intervention is the commitment of these key personnel who have the means to create traction for programs and, at times, can direct funding toward interventions. However, individuals do not work in a vacuum. Existing structures are key elements that make a context more receptive to maintaining interventions. These structures can be formal, such as undergraduate research offices, or informal, such as collaboration between faculty across departments at the university, but in both cases, they provide a venue through which implementation can occur. Finally, alignment to the mission of the university makes institutional uptake and ongoing support more accessible, increasing the likelihood of sustained initiatives.

Last, it is important to note that contextual elements of institutions are not static but *malleable.* Receptive contexts can be developed and maintained to enable effective institutionalization. We found evidence of sites leveraging the BUILD grant to make inroads toward changing institutional conditions. Financial conditions might not be affected by grant funds, but leadership support and personnel commitment were influenced by BUILD, supporting seeking out funding sources that may not already exist within the university. Structurally, several sites created offices to house their interventions. Regarding personnel, people were able to be influenced through training and move into higher leadership positions to gain the resources to support the intervention. The mission of the university is difficult to change; however, evidence can be generated to show how an intervention furthers the goals and mission of the university.

This study’s findings can help current grant-funded programs understand what may be possible at their institutions. While these elements align with prior education literature [45,46], our uniquely coordinated framework offers an understanding of the necessity of considering institutional contexts when shifting from implementation to sustainability. In many STEMM training programs, including the BUILD grants, there is an emphasis on “evidence-based practices.” However, while specific practices may be well-regarded as beneficial, understanding the context in which they can be implemented and ultimately sustained can lead innovators to better invest their energy and finances in the contexts where they are strong or to change contexts to make them more receptive to future programs.

The BUILD experience suggests a vision as to what is possible with a large-scale investment. Understanding what was successful at other institutions also enables program and initiative planners to learn from these successes. This can also influence our perceptions of evidence-based practices: these practices will need to be adapted according to their context and there will not be the same results in all contexts. Further, an understanding of the different contexts can help indicate what may need to change so that an “evidence-based practice” is appropriately designed to fit the institutional context where it would be most effective and *why* those elements may play a role. Understanding *why* can help facilitate *how* to make an intervention more effective [47].

## Conclusion

This study extends the implementation science literature to include institutionalization. Rather than simply identifying what did or did not get implemented, we use implementation science as a means of understanding the contexts that facilitate program sustainability. By understanding what was institutionalized and the contexts that aided or inhibited institutionalization, future innovators, funders, and practitioners can anticipate where investments will likely yield the most benefit and what is necessary for their success. This can help set the foundation for future research on program initiative maintenance both within and beyond higher education and guide future innovations from provisional to a permanent part of the institutional infrastructure.
